# Efficacy and safety of cytoreductive surgery combined with hyperthermic intraperitoneal chemotherapy in patients with pancreatic cancer peritoneal metastasis

**DOI:** 10.1186/s12957-024-03464-9

**Published:** 2024-09-02

**Authors:** Guojun Yan, Kai Zhang, Lijun Yan, Yanbin Zhang

**Affiliations:** Department of Peritoneal Cancer Surgery, Beijing Shijitan Hospital, Capital Medical University, No. 10, Tieyi Road, Yangfangdian Street, Haidian District, Beijing, 100038 China

**Keywords:** Cytoreductive surgery, Hyperthermic intraperitoneal chemotherapy, Peritoneal metastasis, Pancreatic cancer

## Abstract

**Objectives:**

Pancreatic cancer with peritoneal metastasis presents a challenging prognosis, with limited effective treatment options available. This study aims to evaluate the efficacy and safety of combining cytoreductive surgery (CRS) with hyperthermic intraperitoneal chemotherapy (HIPEC) as a treatment strategy for this patient group.

**Methods:**

A retrospective analysis was conducted on patients with peritoneal metastasis of pancreatic cancer who underwent CRS + HIPEC treatment at Beijing Shijitan Hospital from March 2017 to December 2023. The study focused on assessing clinical features, the incidence of sever adverse events (SAEs), and overall survival (OS).

**Results:**

A total of 10 patients were enrolled in this study. The median OS was 24.2 months, suggesting an improvement over traditional therapies. While SAEs were noted, including two cases of severe complications necessitating additional surgical interventions, no perioperative fatalities were recorded. The overall survival time for patients with CC0/1 was not significantly different from that of patients with CC2/3, and no prognostic predictors were identified.

**Conclusions:**

The combination of CRS and HIPEC appears to be a viable and promising treatment modality for patients with peritoneal metastasis of pancreatic cancer, offering an improved survival rate with manageable safety concerns. Further research is needed to refine patient selection criteria and to explore the long-term benefits of this approach.

## Introduction

Pancreatic cancer is associated with a notably poor prognosis and ranks as the 14th most common cancer globally, contributing to the seventh highest number of cancer-related deaths [1, 2]. In China, recent data indicates an incidence of over 13,000 new cases and deaths annually [3]. Despite advancements in healthcare, pancreatic cancer incidence and mortality rates continue to rise [4]. Surgical resection with tumor-free margins (R0) represents the sole opportunity for long-term survival. However, median overall survival (OS) post-surgery and adjuvant chemotherapy ranges from 16.5 to 28.0 months [5, 6]. Challenges such as local recurrence and metastasis are significant impediments to successful treatment.

Approximately half of the patients present with metastatic disease at initial diagnosis, with liver metastases being the most common, followed by peritoneal carcinomatosis, occurring in 9.2–60.0% of cases [7, 8]. Historically considered incurable, peritoneal carcinomatosis from pancreatic origin has been managed palliatively, resulting in a median OS between 1.5 and 17.0 months [7, 9]. Chemotherapy remains the backbone of treatment. Gemcitabine plus nab-paclitaxel has only modestly extended median OS by 1.8 months compared to gemcitabine alone [10], while FOLFIRINOX has shown a slight improvement over gemcitabine monotherapy [11]. Nevertheless, the median OS for metastatic pancreatic cancer hovers around just 4.4 to 11.1 months, irrespective of whether patients receive gemcitabine combination chemotherapy or aggressive regimens like FOLFIRINOX [12]. Regional therapeutic strategies, such as hyperthermic intraperitoneal chemotherapy (HIPEC), have shown limited progress, offering a median overall survival of around 17.0 months [8].

There is a conspicuous gap in the literature concerning the use of a combined approach of cytoreductive surgery (CRS) and HIPEC for the treatment of peritoneal metastases from pancreatic cancer. The efficacy of this approach has been documented in various other peritoneal malignancies [13–16], but its application specifically for pancreatic cancer peritoneal metastasis has not yet been thoroughly explored. This study aims to address this research void by evaluating the safety and efficacy of CRS + HIPEC for managing peritoneal metastases originating from pancreatic cancer.

## Metaerials and methods

The paitents with peritoneal metastasis of pancreatic cancer who received CRS + HIPEC were enrolled at Department of Peritoneal Cancer, Beijing shijitan Hospital, between March 2017 and December 2023. Synchronous peritoneal metastasis was identified by the simultaneous detection of pancreatic cancer and peritoneal metastasis, whereas metachronous peritoneal metastasis was characterized by the occurrence of peritoneal metastasis following pancreatectomy. The research methodology strictly adhered to the ethical guidelines stipulated by the institutional review board, and was consistent with the principles of the 1964 Helsinki Declaration and its subsequent amendments. Approval for the study was granted by the Ethics Committee of Beijing Shijitan Hospital. Written informed consent was obtained from all participants prior to their undergoing CRS + HIPEC, following a detailed explanation of the procedure and its potential risks.

### Inclusion criteria

The decision to pursue CRS combined HIPEC for pancreatic cancer with peritoneal metastasis was informed by a preoperative set of inclusion criteria established to identify candidates who may benefit from this aggressive therapeutic strategy, despite it not being the standard care for this patient cohort. The criteria encompassed: (1) A confirmed diagnosis of pancreatic cancer with biopsy-confirmed or frozen pathology-verified peritoneal metastasis; (2) Assessment of disease extent via imaging modalities, such as computed tomography (CT) or positron emission tomography (PET), suggesting that the peritoneal metastases were potentially resectable; (3) Evaluation of the patient’s overall health and performance status, ensuring suitability for enduring major surgery and its associated risks; (4) Adequate organ function, as determined by laboratory testing, to withstand the potential impact of the procedure; (5) Consensus among a multidisciplinary team, including hepatobiliary and gastrointestinal surgeons, medical oncologists, radiologists, and pathologists, that the patient would likely derive benefit from the CRS + HIPEC approach; and (6) The patient’s informed consent, which entailed an extensive discussion of the procedure’s risks, benefits, and available alternative treatments.

### Procedure of CRS + HIPEC

The CRS + HIPEC procedure involved: (1)Surgical approach: an open abdominal method was adopted for CRS, followed by HIPEC. (2) Tumor burden evaluation: the Peritoneal Cancer Index (PCI) was calculated intraoperatively in accordance with the method described by Sugarbaker, ranging from 0 ~ 39 [17]. (3) Tumor removal: all macroscopically visible tumors, including those involving the viscera and peritoneum affected by carcinomatosis, were resected maximally. The organ resections incorporated procedures on the ascending colon, transverse colon, descending colon, sigmoid colon, complete colon, gastrectomy, small intestine resection, rectal resection, and excision of the ovaries and fallopian tubes, hysterectomy, partial hysterectomy, kidney, and spleen, pancreas, gallbladder, and bladder. The peritonectomy encompassed the bilateral diaphragmatic peritoneum, greater and lesser omentum, bilateral colonic sulcus peritoneum, hepatic round ligament, anterior wall peritoneum, pelvic floor peritoneum, and mesentery. (4) HIPEC Administration: Following the CRS, HIPEC was administered using a combination of docetaxel (120 mg) and cisplatin (120 mg) for a duration of 30 to 60 min. (5) Digestive tract reconstruction: following CRS + HIPEC, digestive tract reconstruction was undertaken. (6) Cytoreduction completeness assessment: the extent of cytoreduction was evaluated based on the size of residual nodules: CC-0 indicated no visible nodules; CC-1, residual nodules smaller than 2.5 mm; CC-2, nodules measuring 2.5 mm to 2.5 cm; and CC-3, nodules larger than 2.5 cm.

### Study endpoints and definitions

Collected clinical data included parameters such as gender, age, Body Mass Index (BMI), Karnofsky Performance Score (KPS). Detailed information pertaining to CRS + HIPEC encompassed operation duration, organ resections, peritoneum resection, anastomosis procedures, specifics of HIPEC administration, PCI score, CC score, and the requirement for blood transfusions, as well as related preoperative and postoperative treatments.

Postoperative complications were graded according to the Clavien-Dindo Classification System, which comprises nine categories with forty-eight adverse events distributed across levels I to IV. Complications at levels III to IV were defined as severe adverse events (SAEs) [18]. Outcome measures were OS, defined as the time from the date of CRS + HIPEC to the last follow-up or death.

### Statistical analysis

All statistical evaluations were conducted employing the SPSS (version 26.0, IBM, Chicago). We opted for the utilization of non-parametric methodologies for the meticulous analysis of the data. OS was analyzed utilizing Kaplan-Meier estimates, with survival curves being determined through the application of the log-rank test. Hazard ratios and *P*-values were calculated using the Cox proportional hazards model.

## Results

**Fig. 1:**
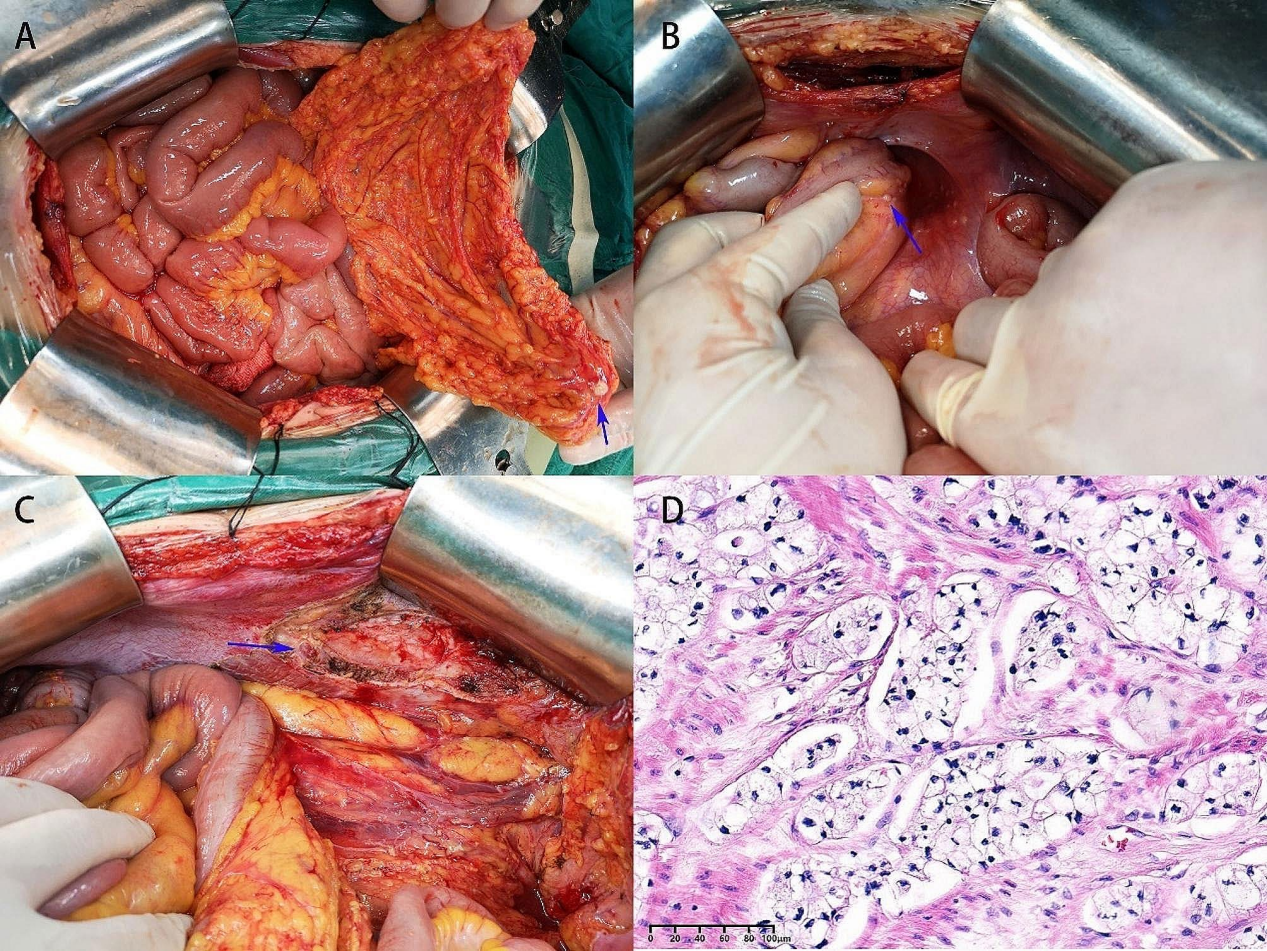
Surgical and histopathological insights. **(A)** Abdominal exploration revealed the greater omentum nodules marked by a blue arrow. **(B)** Detailed view of a suspected lesion in the intestine, indicated by a blue arrow. **(C)** Completion of CRS resulted in the removal of the greater omentum and surrounding tissues, achieving CC-0. **(D)** Histological examination of the excised tissue, using hematoxylin and eosin stain, reveals irregular glandular structures and atypical cells, with a diagnosis of pancreatic duct adenocarcinoma based on cell differentiation levels. Scale bar equals 100 μm

This study enrolled 10 patients, including 3 males and 7 females, with a median age of 52 years (32 ~ 66 years) and a median BMI of 23.55 (19.5 ~ 29.6). The majority had not received treatment before CRS + HIPEC (70%) owing to simultaneous peritoneal metastasis, with pancreatic ductal adenocarcinoma being the most common pathology (60%) (Table 1). Figure 1 showed one case received CRS + HIPEC.

**Table 1:** Characteristics of 10 patients who underwent CRS + HIPEC

Variables	*n*	%
Age [Median]	52 [32 ~ 66]	
Gender		
Male	3	30.0%
Female	7	70.0%
BMI [Median]	24.0 [19.5 ~ 29.6]	
KPS [Median]	90 [80 ~ 100]	
Syn/Met		
Syn	8	80.0%
Met	2	20.0%
Treatment before CRS + HIPEC		
No	7	70.0%
Yes	3	30.0%
Pathology		
Pancreatic ductal adenocarcinoma	6	60.0%
IHC indicated pancreatic origin.	4	40.0%

Met: metachronous; Syn: synchronous; IHC: Immunohistochemistry; BMI: Body Mass Index; KPS: Karnofsky Performance Status

Operative details revealed a median PCI of 19, indicating a diverse spectrum of tumor burdens. The median duration of the CRS + HIPEC procedure was 508 min, with a significant proportion of cases achieving complete cytoreduction (CC-0 to CC-1 in 60% of patients) (Table 2).

Significantly, two patients experienced SAEs (Table 2): one developed a thoracic infection requiring secondary surgery, and another suffered from postoperative intestinal obstruction. Despite these complications, there were no perioperative deaths, underscoring the procedure’s safety profile. Some patients received chemotherapy, targeted therapy, and/or additional surgery following CRS + HIPEC.

**Table 2:** Intraoperative Data and postoperative SAEs for CRS + HIPEC

CRS + HIPEC	All patients (*n* = 10)
PCI, [Median]	19 [9 ~ 38]
Operation time, [Median]	508 min [252 ~ 718 min]
Open HIPEC technique	10
CC0/1	6
SAEs	2

**Fig. 2:**
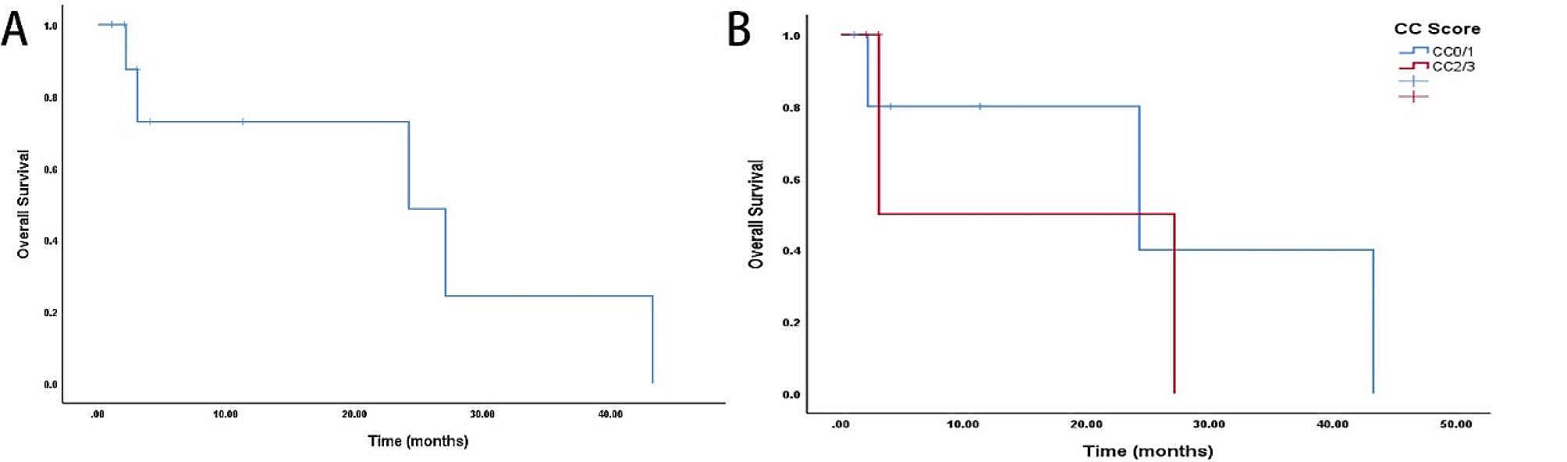
**A**: OS of patients with peritoneal metastasis of pancreatic cancer following CRS+/HIPEC. **B**: No significant differences in OS were observed when comparing patients with peitoneal metastasis of pancreatic cancer in both CC0/1 and CC2/3 groups

The median follow-up period was 12.0 months, during which the median OS reached 24.2 months (Fig. 2), suggesting a potential improvement in outcomes for patients with peritoneal metastasis of pancreatic cancer following CRS + HIPEC. Factors influencing OS as determined by univariate analysis are presented in Table 3. Age, gender, prior chemotherapy, PCI score, CC score, and whether peritoneal metastasis was synchronous or metachronous, along with the postoperative morbidity, were not statistically significant.

**Table 3:** Risk factors analysis

		Univariate Analysis
Prodictor of prognosis	All patients (*n* = 10)	*P*-value
Gender		0.525
Male	3	
Female	7	
Age		0.484
≥ 65	2	
< 65	8	
Prior chemotherapy		0.557
Yes	3	
No	7	
Syn/Met		0.606
Syn	8	
Met	2	
PCI		0.526
> 20	3	
≤ 20	7	
CC		0.543
CC0/1	6	
CC2/3	4	
Postoperative morbidity		0.466
Yes	2	
No	8	

Met: metachronous; Syn: synchronous;

## Discussion

The encouraging findings of our study highlight the potential of CRS + HIPEC as a viable treatment option for pancreatic cancer patients with peritoneal metastasis. The observed median OS of 24.2 months post-treatment marks a significant departure from the historically dismal prognosis associated with this advanced stage of the disease. This improvement in survival metrics suggests that the localized delivery of chemotherapy during HIPEC, combined with the maximal reduction of tumor burden achieved through CRS, can effectively combat the aggressive nature of pancreatic cancer cells within the peritoneal cavity.

CRS + HIPEC was initially applied to the treatment of peritoneal pseudomyxoma in the United States during the 1980s, demonstrating significant therapeutic benefits [19]. Over the ensuing four decades, advancements in technique have expanded the application of this comprehensive approach to a wider array of peritoneal carcinomas. These include metastatic ovarian cancer, colon cancer, gastric cancer, mesothelioma, and pseudomyxoma. Reports on the use of CRS + HIPEC for these indications have been encouraging, highlighting its adaptability and potential as a modality in the treatment of peritoneal malignancies [13, 14, 20, 21]. Furthermore, even in the context of refractory hepatocellular carcinoma, the implementation of CRS + HIPEC has shown promise in improving prognosis, with selected patients experiencing enhanced long-term survival outcomes [22–24].

Conventionally, metastatic disease has been regarded as contraindicative to surgical intervention, owing to the aggressive biological behavior of such tumors. To date, there exists no comprehensive study specifically addressing the role of CRS + HIPEC in the context of peritoneal metastasis of pancreatic cancer. However, a limited number of retrospective analyses and case series have explored the potential benefit of reoperation for recurrent or distant metastatic pancreatic cancer over the past several years. Thomas et al. [25] reviewed patients with recurrent or metastatic disease of pancreatic cancer who underwent secondary surgery and identified that carefully selected cases may derive significant survival benefits from reoperation, with a reported median OS of 36.0 months in lung metastasis or localized recurrence patients. Notably, it appears that lung metastasis may be more amenable to reoperation compared to other sites of disease [25, 26]. For instances of liver metastasis or localized recurrence, median OS following M1 resection was 12.3 months, with 5-year OS rates of 8.1% for liver metastases and 10.1% for distant interaortocaval lymph nodes resection [27]. Shailesh and colleagues also reported on pancreatic cancer patients with metastasis who underwent resection, revealing a median OS of 12.9 months [28]. In a retrospective study conducted by Natasha er al [29], provided important insights into the potential benefits of CRS + HIPEC for patients with peritoneal metastasis of pancreatic cancer. The observation of a 15.0 months OS in one patient following the CRS + HIPEC intervention is noteworthy. Collectively, these studies suggest that judicious patient selection for reoperation may confer substantial survival advantages.

HIPEC has been documented to enhance the penetration of chemotherapeutic agents into tumor tissues, augmenting their cytotoxic effects by several-fold compared to normothermic conditions [8, 30]. The hyperthermic environment itself induces a heat shock response in tumor cells, potentially triggering apoptosis and sensitizing these cells to the cytotoxic effects of chemotherapy [31]. Moreover, preliminary applications of HIPEC specifically in the setting of pancreatic cancer have shown encouraging results [32], suggesting that the combination of surgical resection with HIPEC could yield superior outcomes.

It is important to note that while our study provides evidence supporting the potential benefits of CRS + HIPEC for pancreatic cancer patients with peritoneal metastasis, it also highlights the need for careful patient selection. Further studies are necessary to identify the optimal candidates for this treatment modality and to establish clear guidelines for patient selection.

## Conclusions

The integrated approach of CRS + HIPEC offers a new therapeutic option for patients with peritoneal metastasis from pancreatic cancer. The observed improvements in OS justify further investigation into the role of this combined modality in the management of this challenging disease. Ongoing clinical trials and prospective studies will be instrumental in defining the efficacy, safety, and long-term outcomes of CRS + HIPEC in the treatment of pancreatic cancer.

## Data Availability

No datasets were generated or analysed during the current study.
